# Interstitial Pregnancy: From Medical to Surgical Approach—Report of Three Cases

**DOI:** 10.1155/2018/2815871

**Published:** 2018-10-15

**Authors:** L. Di Tizio, M. R. Spina, S. Gustapane, F. D'Antonio, M. Liberati

**Affiliations:** ^1^Department of Obstetrics and Gynecology, SS Annunziata Hospital, Chieti, Italy; ^2^Department of Medicine and Aging Sciences University “G. d'Annunzio” of Chieti-Pescara, Italy; ^3^Department of Obstetrics and Gynecology, Casa di Cura Salus srl, Brindisi, Italy; ^4^Women's Health and Perinatology Research Group, Department of Clinical Medicine, Faculty of Health Sciences, UiT-The Arctic University of Norway, Tromsø, Norway; ^5^Department of Obstetrics and Gynaecology, University Hospital of Northern Norway, Tromsø, Norway; ^6^“G. d'Annunzio” University, Chieti, Italy

## Abstract

**Background:**

Interstitial pregnancy is a rare form of ectopic pregnancy that usually leads to uterine rupture resulting in sudden life-threatening haemorrhage, need for blood transfusion, and admission to intensive care unit. Mortality rate is 6–7 times higher than that in classical ectopic pregnancy. Uterine rupture has been typically reported to occur at more advanced gestational ages compared to tubal pregnancy although several recent reports have shown a high risk of rupture before 12 weeks of gestation.

**Cases Presentation:**

We report three cases of women affected by interstitial pregnancy, with different clinical symptoms, and managed to be treated with surgery or medical therapy. An emergency laparotomy was performed in the first case by the general surgeon, while in the second case laparoscopy was made by a gynecologist; last case shows the success of systemic administration of methotrexate.

**Conclusion:**

Interstitial pregnancy is still a challenging condition to diagnose and treat; early diagnosis may help to choose the proper management.

## 1. Background

The interstitial part of the fallopian tube is the proximal portion located into the muscular wall of the uterus. Pregnancies implanting in this site are defined as interstitial or cornual [1].

The interstitial part of the tube has a significantly greater capacity to expand before rupture because of its thickness; rupture can result in catastrophic haemorrhage with massive blood loss from the vascular anastomoses between the uterine and the ovarian arteries (Sampson's artery) [2].

Risk factors include assisted reproductive techniques, previous tubal pregnancies, tubal surgeries, a history of pelvic inflammatory disease, and sexually transmitted diseases [3].

Therefore, early detection is crucial to reduce morbidity and mortality.

Surgical approach consists of both laparotomy and laparoscopy techniques; conservative treatment is the administration of systemic methotrexate, checking the serum ß-hCG level on the 0th, the 4th, and the 7th day after, according to the Stovall protocol [4].

## 2. Cases Presentation

Three women were diagnosed with ectopic interstitial pregnancy from 2013 to 2016.

### 2.1. CASE no. 1

A 26-year-old woman (one previous Caesarean section and two previous voluntary terminations), with a history of irregular menstrual cycles, was admitted to the emergency unit with acute abdominal symptoms and vaginal bleeding. She was initially assessed by a general surgeon.

At first assessment, she presented with blood pressure of 70/40 mmHg and severe pallor; abdominal examination revealed guarding and tenderness in both iliac fossae and hypogastrium, while rectal examination revealed pain at the pressure of the pouch of Douglas. At ultrasound scan, corpusculated free fluid was detected. Laboratory test showed haemoglobin level of 10,1 g/dl. At vaginal examination, there was cervical tenderness with fullness in posterior fornix. Transvaginal ultrasound was not performed, since the patient conditions were worsening, and the general surgeon pushed to take the patient to the operating room, even though HCG test and the result of the CT were not ready.

**Figure 1:**
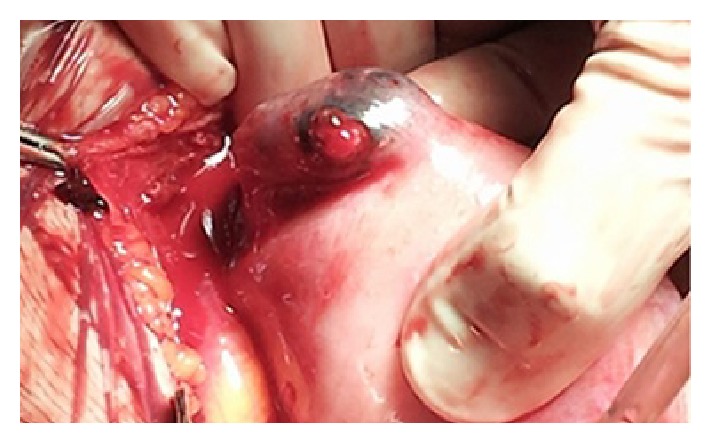


Due to her unstable clinical conditions, the general surgeon decided on emergency laparotomy: subumbilical incision was performed, showing massive hemoperitoneum with blood loss from the uterine angle, due to a ruptured ectopic interstitial pregnancy (Figure 1).

**Figure 2:**
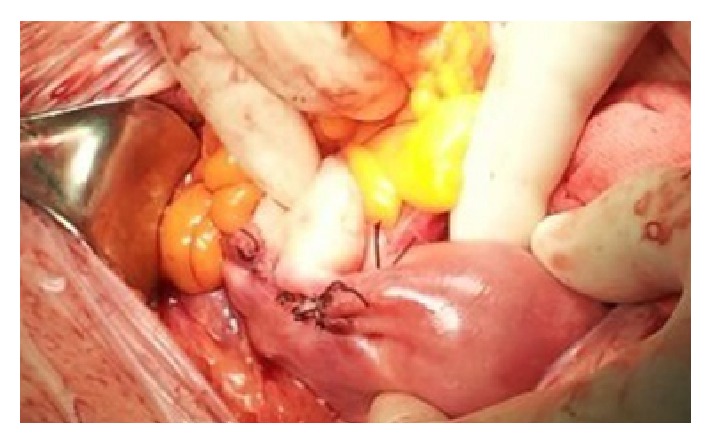


Resection of the cornual area combined with an ipsilateral salpingectomy was performed and a tobacco pouch suture (Figure 2) made to provide an effective haemostasis.

In the operating theatre, multiple arterial blood samples were taken, and a decreasing haemoglobin trend was noticed, with the minimum value of 6,3 g/dl. Plasma expanders, crystalloids, and two blood units were administered. Pregnancy test proved to be positive and CT scan showed massive hemoperitoneum, without detecting any masses in the uterus or adnexal regions.

The following days, serum ß-hCG level showed a decreasing trend (1212,3 UI/L-514,6 UI/L-228,3 UI/L). A week after the discharge, serum ß-hCG level was 8,8 UI/L.

### 2.2. CASE no. 2

The second woman, thirty years old, (one previous voluntary termination) was referred to the emergency obstetrics department with pelvic pain and vaginal bleeding in the past three days.

A blood sample for haemoglobin and ß-hCG levels was taken at admission, while a transvaginal scan revealed the presence of a round mass containing an embryo with positive heart beat located in the isthmic portion of left tube. Furthermore, a small amount of free fluid in the pouch of Douglas was noticed; ß-hCG level at the access was 482,80 UI/L. The patient was haemodynamically stable, without any signs or symptoms of acute abdomen.

**Figure 3:**
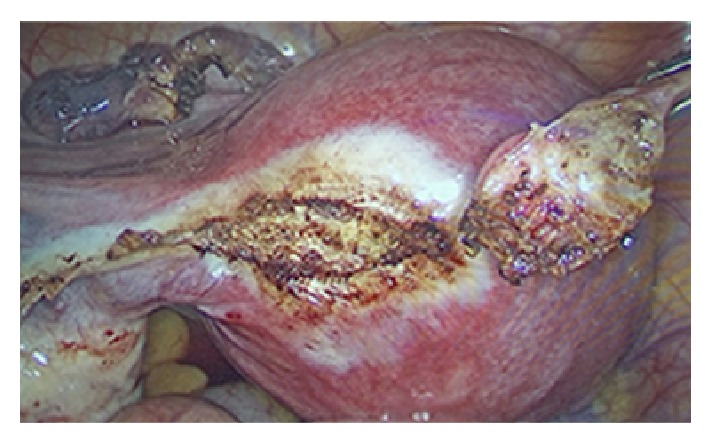


After extensive counselling, elective laparoscopic left salpingectomy was performed (Figure 3) and serum ß-hCG after surgery progressively decreased.

### 2.3. CASE no. 3

**Figure 4:**
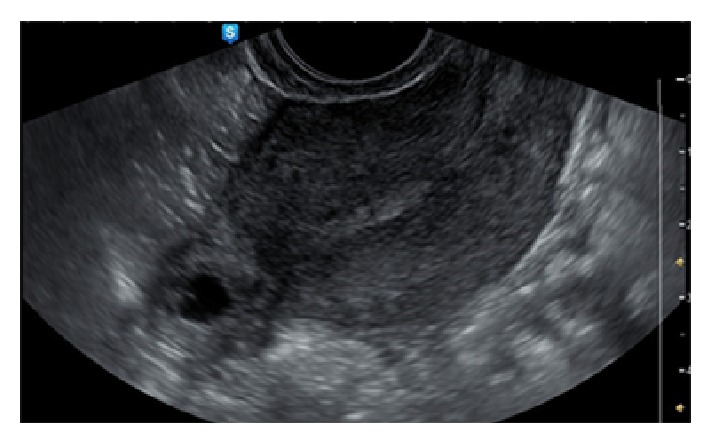


A third woman, thirty eight years old, (tertigravida, nullipara, one previous Caesarean section, and one previous voluntary termination) was admitted to the obstetrics and gynaecologic unit for vaginal blood loss and worsening pelvic pain. Laboratory tests and transvaginal ultrasound scan were immediately performed. The scan revealed the presence of a cornual anechoic mass with a hyperechoic border, compatible with tubal ring in the interstitial portion of the right tube, with maximum diameter of 0,87 cm, (Figure 4) and no free fluid in the pouch of Douglas. At admission, serum ß-hCG level was 3212 UI/L.

In view of the stable clinical conditions and the woman's wish to preserve the tube, intramuscular methotrexate was administered according to Stovall protocol [4]; serum ß–hCG levels were assessed on days 0, 4, and 7. Since the values did not decrease at least 15% between day 4 and day 7, a second dose of methotrexate was administered. ß-hCG levels showed a decreasing trend and became negative after 5 weeks.

## 3. Discussion

Interstitial pregnancy is a rare form of ectopic pregnancy, located in the proximal part of the fallopian tube, which usually leads to uterine rupture with resultant life-threatening hemorrhage [1]. It can be easily misdiagnosed with angular pregnancy; however, the latter is located within the endometrial cavity, in the corner where the tube connects with the fundal part of the uterus [5]. Most ruptures are reported later than in other tubal pregnancies because the myometrium is more distensible than the fallopian tube [1], but this common belief has been dispelled by more recent case series, reporting them at less than 12 weeks [6].

Major risk factors for interstitial pregnancies include assisted reproductive techniques, previous tubal pregnancy, tubal surgery, history of pelvic inflammatory disease, and sexually transmitted diseases [7].

Common symptoms of interstitial pregnancy are abdominal pain and vaginal bleeding, while signs of acute abdomen may occur in case of rupture and hemoperitoneum.

Haemorrhagic shock, due to interstitial pregnancy rupture, occurs in almost a quarter of the cases, thus explaining the relatively high mortality rate of interstitial pregnancies [3]; some authors have reported rupture of the interstitial pregnancy and formation of a hematoma of the broad ligament [8].

Early diagnosis is fundamental to manage this condition safely and it is accomplished using antenatal ultrasound and quantitative HCG assay.

Main diagnostic criteria using transvaginal ultrasound are as follows [9]: an empty uterine cavity, a separate chorionic sac at least 1 cm from the lateral edge of the uterine cavity, and a thin (5 mm) myometrial layer surrounding the gestational sac.

*Ackerman et al.* [10] described the interstitial line sign, which refers to the visualization of an echogenic line that runs from the endometrial cavity to the interstitial region, abutting the interstitial mass or gestational sac.

The paucity of the myometrium around the gestational sac is diagnostic of interstitial pregnancy, while an angular pregnancy has at least 5 mm of myometrium on all its sides [3]; in the first case the embryo is implanted lateral to the round ligament and in the second one embryo is implanted in the lateral angle of the uterine cavity, medial to the uterotubal junction and round ligament [6].

Sonographic findings in two dimensions can be further confirmed using three-dimensional ultrasound, where available, to avoid misdiagnosis with early intrauterine or angular (implantation in the lateral angles of the uterine cavity) pregnancy [11].

Early diagnosis with transvaginal ultrasound allows conservative treatment with methotrexate; if it is made later in gestation, surgical treatment can be required [12].

Traditionally, the treatment of interstitial pregnancy involves hysterectomy or cornual resection, although more conservative approaches have been recently introduced.

Systemic methotrexate is safe and effective, but early recognition is essential. Local injection of methotrexate, either transvaginal or laparoscopic, provides highly effective methods [6].

Recent studies have reported that a pharmacological approach using methotrexate is usually effective, although there is insufficient evidence to recommend local or systemic approach.

Major institution recommends that methotrexate should be the first-line management for hemodynamically stable women with no pain, those with an unruptured ectopic pregnancy, a mass smaller than 35 mm with no visible heartbeat, and a serum b-hCG between 1500 and 5000 IU/l [11].

Other authors have reported an alternative approach by performing a transvaginal ultrasound guided aspiration of the extracelomic fluid from the gestational sac, followed by intrasaccular injection of 25 mg of methotrexate with/without 0.2–0.4 mEq of potassium chloride [11].

The main advantages of local injection of methotrexate include smaller drug dosage, fewer systemic side effects, and higher tissue concentration [6].

Surgical treatment consists of conservative techniques, such as laparoscopic cornual resection, laparoscopic or laparotomic cornuostomy or hysteroscopic removal of interstitial ectopic tissue, and radical operations such as salpingectomy [6, 13] or hysterectomy [6].

Radical surgery is necessary in cases where haemorrhage is life threatening [14].

Due to the abundant blood supply in the cornual region from both uterine and ovarian vessels, rupture occurring after 12 weeks of gestation often leads to severe haemorrhage and eventually maternal death [5].

Laparotomy used to be the preferred surgical approach to the treatment of ruptured cornual pregnancy, especially when it occurs in advanced gestation [6].

Ipsilateral uterine artery ligation should be performed before attempting to repair a ruptured uterine cornu [6] or temporary clipping of the main structures (uterine and ovarian arteries) before the excision [15]; both techniques will help to achieve haemostasis and allow time to repair the cornu [16].

Even in women with ectopic pregnancy with significant hemoperitoneum, laparoscopic surgery has been reported to be safe in experienced hands [14, 17].

Both Tulandi (1995) [18] and Reich (1998) [19] used laparoscopic cornual excision to manage cornual pregnancies. Trends toward less extensive laparoscopic procedures are evident in further case reports using cornuostomy [6].

Most authors agree that the size of cornual gestation determines the best laparoscopic approach; Tulandi [18] reported that salpingostomy is appropriate for gestations of <3.5cm, whereas cornual excision was recommended by Grobman et al. [20] for gestations of >4 cm.

Various endoscopic approaches have been reported, such as electrocauterization, endoloop application, or the encircling suture before evacuation of the conceptus [21].

In a retrospective study, Moon et al. [21] reported that “the endoloop and the encircling suture methods are simple, safe, effective, and nearly bloodless.” He also described a technique which uses highly diluted vasopressin for hemostasis during laparoscopic surgery (1 ampoule [20 U] of vasopressin diluted in 1000 ml of normal saline [1000-fold] and 150–250 ml [0.02 U/ml] of diluted vasopressin injected in the uterus below the interstitial pregnancy [22]. There were no uterine ruptures in the pregnancies following these methods of endoscopic management.

Other authors described other techniques such as the “purse-string” technique, a haemostatic suture passed at the base of the mass before removing it [13], or a sort of square knot at the border of the cornuostomy [23].

Hysteroscopic management can be particularly suitable for women who are reluctant to undergo medical treatment with methotrexate or in whom this treatment fails or is unavailable. Minelli et al. stated that, in expert hands, resection of the corneal endometrium, including the tubal ostium, can be successfully performed without perforation of the uterus [6].

Surgical treatment involving resection of the cornual region is associated with decreased fertility and increased uterine rupture rates in future pregnancies [24].

It is also described for uterine rupture in a pregnant woman with previous surgery of cornuostomy [25].

One of the concerns of future pregnancy is rupture of the interstitial portion of the tube (uterine rupture). The postulated mechanism is through a defective area of uterine wall [26].

Some authorities suggest suturing the uterine wall after surgical management to reinforce the defective area in the uterine wall. Term deliveries have, however, been reported following laparoscopic treatment of corneal pregnancy without reinforcing sutures [27, 28].

Most authors agreed that Caesarean section should be the optimum mode of delivery for all pregnancies following cornual pregnancy [14].

The second concern after conservative management of cornual pregnancy is recurrence of ectopic pregnancy, particularly cornual pregnancy on the same side [6].

Thus, conservative treatment should be the first treatment option if the patient is stable and there is the aim to preserve fertility; otherwise interstitial pregnancy should be removed via laparoscopy or laparotomy.

However, more cases and studies should be reported; limitations of our case report study are based on the paucity of the patients.

## 4. Conclusion

Cornual pregnancy poses a significant diagnostic and therapeutic challenge; early diagnosis may help to choose the proper management and treatment; according to the clinical presentation and the haemodynamic stability, it should be based on laboratory exams (serum ß-hCG level) and transvaginal ultrasound.

## Abbreviations

ß-hCG: The *β*-subunit of human chorionic gonadotropin
CRL: Crown-rump length
3D scan: Three-dimensional scan
hCG: Human chorionic gonadotropin.
